# Individuals with prediabetes identified by HbA1c undergoing coronary angiography have worse cardiometabolic profile than those identified by fasting glucose

**DOI:** 10.1186/1758-5996-6-138

**Published:** 2014-12-13

**Authors:** Valdecira M Piveta, Celia S Bittencourt, Carolina SV Oliveira, Pedro Saddi-Rosa, Deyse M Meira, Fernando MA Giuffrida, André F Reis

**Affiliations:** Diabetes Center, Endocrinology Unit, Universidade Federal de São Paulo, São Paulo, Brazil; Departamento de Ciências da Vida - Colegiado de Medicina, Universidade do Estado da Bahia, Rua Silveira Martins, 2555 Cabula, Salvador, BA CEP: 41.150-000, Brazil; Centro de Endocrinologia do Estado da Bahia (CEDEBA), Salvador, Brazil

**Keywords:** Cardiovascular disease, Hyperglycemia, Coronary angiography, Type 2 diabetes, Prediabetes, Hemoglobin A1c

## Abstract

**Background:**

Type 2 diabetes mellitus has well known deleterious effects on coronary artery disease (CAD). The role of milder hyperglycemic states such as prediabetes (PD) on CAD is debatable. Glycated hemoglobin (HbA1c) has recently been advocated as a diagnostic tool for diabetes mellitus (DM) and PD. This study aims to assess the cardiometabolic risk profile and coronary lesions of patients with PD undergoing coronary angiography identified either by fasting plasma glucose (FPG) or HbA1c levels.

**Methods:**

We studied 514 individuals without previously known glucose disturbances. Their glycemic status was assessed by FPG and HbA1c (HPLC) and classified according to ADA guidelines, using each parameter independently, as having normal glucose tolerance (N), PD, or DM. CAD was defined as stenosis greater than 50% in one major coronary vessel or branch. Framingham score was calculated.

**Results:**

Subjects with PD had a similar frequency of CAD compared do N individuals by both FPG (61 vs. 59.3%) and HbA1c (55.4 vs 61.2%) (p non-significant for linear-by-linear association). PD individuals identified by FPG had worse HOMA2B (mean [95% CI] 65.4 [60.9-69.9] vs. 76.6 [71.4-81.9]) and HOMA2-IR (1.10 [0.98-1.22] vs. 0.80 [0.72-0.89]) when compared to N controls. PD individuals identified by HbA1c had higher frequency of Framingham risk above 20% (25.4 vs 11.8%), arterial hypertension (87.8 vs 72.6%), and dyslipidemia (83.8 vs 72%) compared to N individuals. PD associated with an increased number of coronary lesions only when diagnosed by HbA1c (median [interquartile interval] 2 [0–4] PD versus 1 [0-3.75] N, p = 0.03 for trend).

**Conclusions:**

HbA1c was more effective than FPG in identifying individuals with PD associated with high cardiovascular risk profile in a sample of individuals undergoing coronary angiography.

## Background

It is well established that patients with type 2 diabetes mellitus have a higher risk of developing atherosclerosis and cardiovascular disease (CVD) than non-diabetic individuals. This condition accounts for the majority of deaths in individuals with diabetes mellitus (DM) [1]. Acceleration of atherosclerosis in the diabetic milieu is supposed to be related to different mechanisms such as insulin deficiency, insulin resistance, and other metabolic alterations usually associated with type 2 diabetes like arterial hypertension and dyslipidemia [2, 3]. These classical risk factors, including glycemic control itself, are associated with both presence and severity of coronary artery disease (CAD) in type 2 diabetes [4].

Undiagnosed hyperglycemia is a common finding in acute coronary syndromes and active search for this disorder in the inpatient population is warranted [5]. Also, several studies demonstrated that a large proportion of patients submitted to elective coronary angiography have alterations in glucose levels such as type 2 diabetes and prediabetes (PD), most of them being unaware of their glycemic status. The role of milder hyperglycemic states such as PD on CAD remains largely unknown. Some studies suggest that individuals with PD have an unfavorable metabolic profile with more cardiovascular risk markers, being at increased risk of type 2 diabetes and CAD [6, 7].

Moreover, there is still a strong debate whether individuals with PD identified by different parameters would present different metabolic patterns and related cardiovascular risk. In this regard, we have several tests to identify PD such as fasting plasma glucose (FPG), oral glucose tolerance test (OGTT), and more recently the use of hemoglobin A1c (HbA1c) levels [8]. HbA1c has a poor correlation with FPG, but there is an ongoing debate in literature about which one identifies individuals at a higher cardiometabolic risk and at the same time suffers less diagnostic interference from CAD itself [7–9]. The importance of this discussion lies on the possibility of identifying individuals at increased risk of CVD in order to offer more specific preventive measures.

In this study we aim to assess whether the frequency of CAD, its severity, and cardiovascular risk markers are different in patients with PD diagnosed by FPG and HbA1c classification criteria as compared to type 2 diabetes and normoglycemic subjects.

## Methods

A total of 813 consecutive patients who underwent coronary angiography at Hospital São Paulo (at the Federal University of São Paulo) were enrolled in the present study. Individuals with previously diagnosed DM were excluded, resulting in 514 eligible patients. Study subjects, individually and/or as a group, had mixed ethnic background (African, Amerindian, Asian, and European Caucasian of several different countries of origin), reflecting the heterogeneity of the Brazilian population [10].

A blood sample was drawn before coronary angiography, after an overnight fast for analysis of FPG, plasma insulin, HbA1c (HPLC, reference value: 4.5-6.0%), lipid profile (total serum cholesterol, LDL-cholesterol, HDL-cholesterol, and serum triglycerides), TSH, and creatinine levels. Glomerular filtration rate was estimated using the Modification of Diet in Renal Disease (MDRD) study equation [11]. Insulin resistance and beta-cell secretion were estimated by HOMA2-IR and HOMA2B indexes respectively, calculated by previously described methods [12].

Patients were referred to coronary angiography by their physicians for various reasons, including presence of stable angina, a positive stress test, or for preoperative evaluation for cardiac valvular disease or peripheral vascular disease surgeries. Clinical data as well as personal and family history of cardiovascular disease, DM, and associated diseases were obtained by interview and medical examination by one member of the research group. Patients had their glycemic statuses assessed by FPG and HbA1c levels. They were classified by both criteria according to ADA guidelines in normal (N), PD, and DM [8]. For the purpose of this investigation, CAD was defined as any stenosis >50% in at least one major coronary vessel or branch. We further computed the number of significant coronary lesion (stenosis greater than 50% on angiography), as a parameter to verify the extent and severity of visible CAD [4].

Arterial hypertension was defined as systolic blood pressure (SBP) ≥ 140mmHg and/or diastolic blood pressure (DBP) ≥ 90mmHg, or SBP and DBP below these values in the presence of antihypertensive medication and history of arterial hypertension. Subjects were considered to have dyslipidemia either if levels of LDL-cholesterol were ≥160mg/dL, HDL-cholesterol below 40mg/dL in men and 50mg/dL in women, triglycerides >200mg/dL, or in the presence of use of lipid lowering medications (statin/fibrates) [13].

The Framingham score was calculated as described elsewhere [14]. In brief, gender, age, LDL-cholesterol levels, HDL-cholesterol levels, SBP, DBP, presence of DM, and current smoking status were used to calculate the 10-year risk of CAD. Individuals with calculated risk above 20% were considered as high risk for coronary events [15]. All participants gave written informed consent. The study was approved by the ethics committee of UNIFESP.

Besides previous DM, exclusion criteria included a creatinine clearance lower than 50 mL/min, abnormal thyroid function, the presence of active inflammatory disease or neoplasia, or anemia (Hemoglobin < 10 g/dL), conditions that could potentially interfere in some of of the laboratory measurements performed or in the presence of CAD itself. Patients with acute coronary syndromes and ST segment elevation have been excluded because thrombotic events could overestimate stenosis.

Comparisons of continuous variables among normal, PD, and DM groups were made by ANOVA. Means and 95% confidence intervals (CI) were recorded, instead of means + SD, to enable comparisons between individuals classified by both FPG and HbA1c, since these don’t represent different subgroups, but the same individuals grouped differently. Number of coronary lesions was assessed by Jonckheere-Terpstra test, p values for trend were recorded. Categorical variables were assessed by Mantel-Haenszel test and p for linear-by-linear association was recorded. A two-tailed value of p < 0.05 was considered statistically significant.

## Results

When classified by the FPG criterion, individuals with PD had similar demographic and clinical characteristics such as age, BMI, abdominal circumference, blood pressure, and lipid levels compared to other groups. They had higher FPG and HbA1c levels (by definition), as well as higher insulin resistance and lower beta-cell function assessed by HOMA indexes, when compared to N individuals. DM individuals had only higher FPG and HbA1c levels, being also more insulin resistant when compared to both N and PD individuals. Beta-cell secretion of these individuals was similar to the other groups (Table 1). No differences among the three groups divided according to the FPG criterion were observed regarding the presence or total number of coronary lesions and frequencies of individuals having Framingham Risk Score above 20%, arterial hypertension, and dyslipidemia (Table 2).

**Table 1:** **Clinical and laboratory features of individuals classified according to FPG levels**

	Classification according to fasting plasma glucose		
	Normal (FPG < 100 mg/dL)	Prediabetes (FPG 100–125 mg/dL)	Diabetes (FPG ≥ 126 mg/dL)
n (%)	271	210	31
**Age (years)**	59.0 (57.7-60.4)	59.5 (58.2-60.9)	59.0 (56.2-60.2)
**Weight (kg)**	72.0 (70.2-73.8)	72.87(70.8-74.6)	77.2 (71.0-83.4)
**BMI (kg/m** ^**2**^ **)**	25.9 (25.2-26.6)	26.4 (25.6-27.1)	28.8 (26.6-27.1)
**Abdominal circumference (cm)**	96.0 (94.1-97.6)	97.4 (95.7-99.1)	98.9 (93.3-104.6)
**SBP (mmHg)**	134.4 (131.8-137.0)	140.0 (136.6-143.4)	138.3 (130.7-145.9)
**DBP (mmHg)**	78.3 (76.5-80.0)	82.4 (80.2-84.6)	81.7 (76.3-87.0)
**FPG (mg/dL)**	91.2 (90.5-91.9)	108.6 (107.8-109.5)*	141.3 (135.3-147.4)**
**HOMA2B**	76.6 (71.4-81.9)	65.4 (60.9-69.9)*	68.7 (53.4-84.0)
**HOMA2-IR**	0.80 (0.72-0.89)	1.10 (0.98-1.22)*	2.49 (1.69-3.29)**
**HbA1c (%)**	5.76 (5.70-5.81)	5.93 (5.86-5.99)*	6.65 (6.30-6.99)**
**HbA1c (mmol/mol)**	39.3 (38.7-39.9)	41.2 (40.4-41.8)*	49.1 (45.3-52.8)**
**LDL (mg/dL)**	100.5 (96.1-104.9)	108.9 (103.8-114.0)	101.8 (87.8-115.9)
**HDL (mg/dL)**	41.0 (39.5-42.7)	41.0 (39.4-42.5)	42.0 (36.2-47.8)
**Triglycerides (mg/dL)**	136.4 (126.4-146.3)	140.9 (131.0-150.7)	155.9 (123.0-188.8)

Values expressed in means (95% CI). *p < 0.05 vs. Normal; **p < 0.05 vs. Normal AND Prediabetes.

**Table 2:** **Coronary lesions and cardiovascular risk factors of individuals classified according to FPG levels**

	Classification according to FPG			
	Normal	Prediabetes	Diabetes	p
n	271	210	31	
**Total coronary lesions**	2 [0–4]	2 [0–4]	2 [0–4.25]	NS
**Framingham risk ≥ 20% (%)**	15.1	25.4	6.9	NS
**CAD (stenosis > 50%) (%)**	59.3	61.0	65.6	NS
**Hypertension (%)**	80.5	86.2	87.5	NS
**Dyslipidemia (%)**	80.7	82.1	71.9	NS

When classified by the HbA1c criterion, individuals with PD had also similar demographic and clinical characteristics such as age, BMI, abdominal circumference, blood pressure, and lipid levels when compared to the other two groups. They had higher glycemic parameter levels (also by definition), but HOMA indexes were not different between PD and N. DM individuals had higher FPG and HbA1c levels, and were more insulin resistant than both N and PD (Table 3). Regarding cardiovascular risk markers, total number of coronary lesions, Framingham Risk Score above 20%, arterial hypertension, and dyslipidemia showed a linear and positive association with N, PD, and DM categories divided by HbA1c (Table 4). Frequencies of individuals utilizing antihypertensive or lipid-lowering medication were similar between groups, regardless of using FPG or HbA1c as the grouping criterion.

**Table 3:** **Clinical and laboratory features of individuals classified according to HbA1c levels**

	Classification according to HbA1c		
	Normal (HbA1c < 5.7%)	Prediabetes (HbA1c 5.7-6.4%)	Diabetes (HbA1c ≥ 6.5%)
n (%)	168	279	67
**Age (years)**	57.4 (55.6-59.2)	59.8 (58.6-61.0)	61.5 (59.3-63.7)
**Weight (kg)**	73.4 (71.1-75.7)	71.7 (70.1-73.4)	74.6 (70.3-78.8)
**BMI (kg/m** ^**2**^ **)**	26.1 (25.0-27.1)	26.0 (25.4-26.7)	27.6 (26.1-29.0)
**Abdominal circumference (cm)**	96.8 (94.9-98.6)	96.0 (94.4-97.6)	99.9 (96.5-103.3)
**SBP (mmHg)**	135.4 (131.8-139.0)	138.0 (135.2-140.7)	137.0 (130.7-143.3)
**DBP (mmHg)**	81.2 (79.0-83.5)	79.8 (77.9-81.6)	79.4 (75.3-83.5)
**FPG (mg/dL)**	97.0 (95.1-98.8)	100.6 (99.2-102.0)*	115.9 (110.3-121.4)**
**HOMA2B**	68.6 (63.1-74.0)	72.1 (67.6-76.7)	75.5 (62.7-88.2)
**HOMA2-IR**	0.82 (0.70-0.94)	0.99 (0.88-1.09)	1.43 (1.13-1.74)**
**HbA1c (%)**	5.31 (5.27-5.35)	5.99 (5.97-6.02)*	6.85 (6.72-6.98)**
**HbA1c (mmol/mol)**	34.4 (34.0-34.8)	41.8 (41.6-42.2)*	51.3 (49.9-52.7)**
**LDL (mg/dL)**	100.6 (95.5-105.8)	106.5 (102.0-111.0)	101.0 (90.4-111.5)
**HDL (mg/dL)**	40.9 (38.8-42.9)	41.5 (40.1-42.9)	40.3 (36.8-43.8)
**Triglycerides (mg/dL)**	138.8 (125.8-151.8)	136.9 (128.1-145.8)	148.8 (127.1-170.4)

Values expressed in means (95% CI). *p < 0.05 vs. Normal; **p < 0.05 vs. Normal AND Prediabetes.

**Table 4:** **Coronary lesions and cardiovascular risk factors of individuals classified according to HbA1c levels**

	Classification according to HbA1c			
	Normal	Prediabetes	Diabetes	p
n	168	279	67	
**Total coronary lesions**	**1 [0–3.75]**	**2 [0–4]**	**2 [0–5]**	0.03*
**Framingham risk ≥ 20% (%)**	**11.8**	**25.4**	**26.2**	0.006**
**CAD (stenosis > 50%) (%)**	55.4	61.2	63.6	NS
**Hypertension (%)**	**72.6**	**87.8**	**89.6**	< 0.001**
**Dyslipidemia (%)**	**72.0**	**83.8**	**90.9**	< 0.001**

*p for trend, Jonckheere-Terpstra test; **p for linear-by-linear association, Mantel-Haenszel test.

## Discussion

Our study suggests that the use of HbA1c for the diagnosis of PD identifies individuals with more cardiovascular risk factors than the use of FPG in a high-risk population of individuals undergoing coronary angiography, as assessed by the Framingham score. PD individuals diagnosed by HbA1c also presented more severe CAD when compared to N subjects. This profile was not seen in PD individuals identified by FPG.

As expected, glycemic variables and HOMA indexes were different in individuals divided by FPG, since this measurement itself is used in the HOMA calculations. Interestingly, even though they demonstrated worse HOMA2B and HOMA2-IR as compared to normoglycemic subjects, individuals with PD did not show other differences in cardiometabolic risk profile. In the identification of hyperglycemia, the use of FPG, 2-h postload glucose, and HbA1c are associate with distinct patterns of insulin sensitivity and insulin secretion, which could possibly explain these findings [16]. For example, PD identified by FPG is related to both hepatic insulin resistance and reduction in first-phase insulin secretion, leading to excessive fasting hepatic glucose production. Individuals with impaired glucose tolerance (IGT) have muscle insulin resistance along with defective late insulin secretion [17]. HOMA2B and HOMA2-IR were not different between PD and normoglycemic subjects identified by HbA1c, since HbA1c levels possibly results form a variable combining mechanisms related to both impaired fasting glucose (IFG) and IGT [18]. In our study, DM individuals identified by HbA1c were more insulin resistant than both N and PD individuals as expected, nonetheless they had higher beta-cell secretion compared to the N group. This finding could be possibly explained by a compensatory insulin secretion mechanism in the initial phases of type 2 DM, since individuals were all recently diagnosed.

Regarding cardiovascular risk factors, Di Pino et al. found that PD according to HbA1c levels selected individuals with higher cardiovascular risk compared to those with PD detected by either FPG or OGTT [19]. Our findings of no difference in CV risk among patients grouped according to FPG are compatible with this previous study. Among PD individuals identified by HbA1c, there was a higher proportion of PD individuals having Framingham risk above 20%, as well as more dyslipidemia and arterial hypertension when compared to normoglycemic subjects. These differences were not observed in the comparison of PD and normoglycemic individuals diagnosed using FPG. Of note, lipid levels, SBP, DBP, and prevalence of coronary lesion were not different between groups, neither with FPG nor with HbA1c (Tables 1, 2, 3 and 4). As our study group is composed of outpatients with high cardiovascular risk referred to coronary angiography, this could possibly explain the small difference between subgroups. Furthermore, the validity of the Framingham score for predicting future coronary events in this kind of population could be questioned, since it has not been designed with this goal.

There is an ongoing controversy about whether FPG or post load glucose would have a stronger association with atherosclerosis, and also about which kind of intervention regarding these parameters would modify cardiovascular risk more effectively [17, 20]. The question about the use of HbA1c for stratification of cardiovascular risk in PD subjects is under investigation. Di Pino et al. found that PD diagnosed according to HbA1c levels selected individuals which demonstrated more markers of atherosclerosis such as increased intima-media thickness (IMT) than normoglycemic subjects [19]. Similar results were found in non-diabetic Chinese subjects, in whom HbA1c demonstrated a more robust association with aortic arterial stiffness than fasting or 2-h post load glucose levels [21]. On the other hand, compared with subjects with PD identified by HbA1c only, IGT-only individuals exhibited significantly more classical and non-classical cardiovascular risk markers, such as higher SBP and C-Reactive Protein (CRP), as well as lower HDL-cholesterol and insulin sensitivity, resulting in a higher Framingham score for 10-year CVD risk and subclinical atherosclerosis as assessed by carotid IMT. However, authors observed no differences in cardiometabolic risk profile among PD subgroups diagnosed by FPG only, HbA1c only, and by both HbA1c and FPG [18]. In a German study with 8,365 individuals, HbA1c diagnosed individuals with PD with more cardiovascular risk factors, but not a higher frequency of CVD [22]. Another study of 2,076 individuals without DM showed HbA1c to be independently associated with progression of coronary artery calcification in 5 years [23].

Moreover, the use of HbA1c for the diagnosis of dysglycemia is more recent. Most studies that have addressed the issue of PD and CVD have used fasting glucose and/or OGTT as definitions of diagnosis. For example, the Australian Diabetes, Obesity, and Lifestyle Study (AusDiab) showed that the risk of death was increased in those with IFG and IGT, but the risk of CVD mortality was only significantly higher for those with IFG, but not IGT, compared with normal glucose tolerance, even after adjustment for age, sex, and other traditional CVD risk factors [20]. The WOSCOPS study showed that IFG is not a good risk marker for CVD, in 6447 middle-aged white male population over 15 years of follow-up [24]. Furthermore, one systematic review investigated the relative risk (RR) for CVD associated with IFG and IGT and found only a modest increase in the risk for CVD with both PD states [6]. In the Diabetes Prevention Program (DPP), those subjects with IFG and/or IGT that achieved normal glucose regulation or received specific medical treatment for CVD risk factors significantly reduced the estimated risk assessed by the Framingham Risk Score [25].

The use of HbA1c measurements have some advantages over assessments of both FPG or OGTT. Some of them are very practical concerning patients submitted to coronary angiography, since no fasting is necessary and there is less interference during periods of stress [26]. Among the possible explanations for our finding of individuals with PD identified by HbA1c having higher cardiovascular risk than those identified by FPG, we could speculate that lower levels of HbA1c are more closely related to postprandial glucose than to FPG levels. In this regard there are some suggestions that postprandial glucose in the non-diabetic range is a better marker of CVD risk [27]. However, this was not confirmed by the Emerging Risk Factors Collaboration study, where the improvement in the prediction of CVD given by assessment of FPG, postprandial glucose, and even HbA1c levels were similar. This large prospective study involving more than 294,000 subjects without CVD at baseline argues against good incremental benefit for prediction of CVD with the use of HbA1c [28]. The same conclusion was found by Schöttker and co-authors, who showed that in Germans cardiovascular risk prediction did not improve by adding either FPG or HbA1c in individuals without diabetes (except for a potential slight improvement in men with HbA1c) [22].

Our study has several limitations. Firstly, we studied individuals with high cardiovascular risk profile, as they had clinical indication for cardiac catheterization. Therefore, our data are not applicable to the general population. In the same way, there are differences in the hemoglobin glycation among ethnic groups, which could lead to discrepancies in the correlation between HbA1c and FPG levels [29]. Thus the diverse ethnic background of the Brazilian population could influence this confounder [10]. Secondly, another important limitation is the fact that OGTTs were not performed. We are aware that the 2-h glucose postload value which allows diagnosis of glucose intolerance (PD) is a good marker of CVD, and may be a good test to identify higher risk cardiovascular profile [20]. Thirdly, the 50% stenosis criterion utilized to regard CAD as significant could be a potential source of bias, since thrombotic events can occur in patients bearing thinner but unstable plaques. Finally, an important limitation of our study is the cross-sectional design, precluding any causal interpretations of associations between PD and the risk of CAD.

## Conclusions

HbA1c identified individuals with PD bearing a worse cardiometabolic risk profile as assessed by the Framingham score and more severe CAD when compared to either normoglycemic subjects or subjects with PD identified by FPG, in a sample of high cardiovascular risk individuals.

## Abbreviations

CAD: Coronary artery disease
CVD: Cardiovascular disease
DBP: Diastolic blood pressure
FPG: Fasting plasma glucose
HDL: High-density lipoprotein
HOMA: Homeostasis model assessment
LDL: Low-density lipoprotein
OGTT: Oral glucose tolerance test
PD: Prediabetes
SBP: Systolic blood pressure.
